# Psychological effects of low intensity conflict (LIC) operations

**DOI:** 10.4103/0019-5545.31553

**Published:** 2006

**Authors:** Suprakash Chaudhury, D.S. Goel, Harcharan Singh

**Affiliations:** * (Retd) Professor and Head, Department of Psychiatry, Ranchi Institute of Neuropsychiatry and Allied Sciences (RINPAS). Formerly HOD, Psychiatry Department, Command Hospital (Northern Command); ** (Retd) Formerly Senior Adviser (Psychiatry), Armed Forces Medical Services and National Consultant Mental Health; *** (Retd) Formerly Commandant, Command Hospital (Northern Command) and Director General Medical Services (Army)

**Keywords:** Low intensity conflict, psychological morbidity, depression, alcoholism, fatigue

## Abstract

**Background:**

A burgeoning clinical and empirical literature has provided incontrovertible evidence that combat operations exact a heavy toll in terms of human suffering not only on combatants but also military support personnel. Though the Indian army is engaged in low intensity conflict (LIC) operations for over five decades, the psychological effects of LIC deployment on soldiers have not been adequately studied.

**Aims:**

To evaluate the psychological effects of deployment in LIC operations on service personnel.

**Methods:**

Five hundred and sixty-eight servicemen engaged in LIC operations and equal number of age- and rank-matched personnel in adjoining peace areas were evaluated with a self-made questionnaire, General Health Questionnaire (GHQ), Carroll Rating Scale for Depression (CRSD), State-Trait Anxiety Inventory (STAI), Michigan Alcoholism Screening Test (MAST), Impact of Events Scale (IES), Perceived Stress Questionnaire (PSQ), Multidimensional Fatigue Inventory (MFI), Hindi PEN inventory, Satisfaction With Life Scale (SWLS) and Locus Of Control (LOC) scale.

**Results:**

Respondents from LIC area had significantly higher scores on CRSD, MAST, GHQ, IES, and general fatigue, physical fatigue, and mental fatigue subscale of the MFI in comparison to those located in other areas. Significantly higher number of respondents from highly active LIC and with more than one-year service in LIC scored above cut-off levels on CRSD, MAST and GHQ.

**Conclusions:**

The psychological status of troops was directly related both to the duration of stay and the nature of LIC area.

## INTRODUCTION

Low intensity conflicts (LIC) are territorially limited politico-military struggles to achieve political, social, economic or psychological objectives. LIC is often characterized by limitations of armaments, tactics and levels of force. They are often protracted and involve military, diplomatic, economic and psychological pressure through terrorism and insurgency. Troops trained in conventional warfare experience significant stress in such LIC operations.^1^,^2^ Conventional military training makes the soldier think in clear-cut extremes like black and white, friend and foe. This tendency often leads to problems in LIC where the concept of ‘enemy’ cannot be applied to one's own people.^1^,^2^ The contributory factors, which increase the stress level on soldiers participating in LIC, are the product of a complex interplay of three elements involved—the militant, the local population and the soldier.^2^ The development of militancy often has its roots in the regional aspirations of a people governed by an insensitive, unresponsive and corrupt administration. Since the violent acts of militancy appear to offer, at least initially, a quick solution to complex problems, they hold a special attraction to the youth who are often unemployed and frustrated.^2^ Criminals and lumpen elements then join in. The local population tends to think that they have been wronged by the administration. They tend to look at the militant as their ‘own boy’ fighting for a just cause, and the security forces as the long and cruel hand of the administration, particularly when there are human rights violations.^2^ Propaganda by neighboring countries and inter-national agencies may further alienate the local population. In this background the soldier, often from a different cultural milieu, is looked upon as an outsider.^2^ The security forces thus end up fighting an elusive enemy, in the absence of any reliable intelligence, and lack of cooperation or even active resentment of the local population.^2^ Ambiguity of aim, lack of visible success, high casualty rates tend to erode morale among security forces.^1^,^2^ Several operational factors such as fatigue, unpredictability of threat, extended tenures of stay, absence of recreational avenues, domestic worries, irregular mail, problems related to leave and railway travel increase the level of frustration.^1^,^2^

In conventional operations of war, on the other hand, the battle lines are clearly drawn. The enemy is clearly identifiable and aggression can be unequivocally channeled in his direction. Organizational as well as national goals are clear and unambiguous, public support is assured and the soldier comes to regard himself as a living symbol of patriotic pride. Units operate from a firm base where relaxation in a relatively safe environment is readily accessible and lines of communication are secure. Limited periods of intense stress followed by adequate recovery phases do not significantly sap the psychological resources of the soldier in such formal, structured combat scenarios unless the operations are unduly prolonged or are attended by repeated reverses.^1^ The situation in LIC is diametrically opposite. Prolonged spells of stress punctuated by quantitatively and qualitatively inadequate opportunities for rest and relaxation impose immense and often unbearable demands on even otherwise robust subjects. This may result in psychological distress, combat stress disorder or post-traumatic stress disorder (PTSD). In addition, overstaying leave, desertion, abuse of alcohol or drugs, suicide, and cases of soldiers running ‘amok’, shooting at their superiors and colleagues may be symptoms of a serious malady plaguing the troops.^3^,^4^

In stark contrast to the plethora of Western literature on combat-related stress and its consequences,^5^–^16^ only sparse Indian works exist. Goel^1^ using a self-made questionnaire highlighted the determinants of motivation and morale of soldiers in LIC. The psychological effects of LIC were not the focus of this study. Soldiers in LIC environment experienced a number of stressful events including operation stressors, domestic stressors, intra-unit hassles, physical and situation attributes of operation zone, and sociopolitical stressors.^17^ Troops deployed in LIC had significantly higher psychiatric morbidity, alcohol use, unfavourable response to task, diminished efficiency, frustration, maladjustment, tension, isolation, etc.^18^ The failure to use standardized psychological scales was a major weakness of these early studies. Chaudhury^19^ reported high psychiatric morbidity, depression and alcoholism in soldiers in LIC, but the study lacked a control group. The present work was undertaken in this context.

## METHODS

A multidimensional approach was adopted to acquire data for the study. There were visits to forward areas to assess ground realities and to interact with officers and troops deployed there. The subjects of the study consisted of 568 officers, junior commissioned officers and other ranks randomly selected from units deployed in LIC. Equal number of age-, sex- and rank-matched subjects posted in nearby locations but not deployed in LIC formed the control group. None of the subjects had a past or family history of psychiatric disorders. All subjects gave informed consent. To ensure confidentiality the subjects were not required to fill identifying personal data in the questionnaire. After explaining the aims of the study and assuring full confidentiality, all subjects were administered a self-made personal questionnaire and the following self-rating scales administered in group setting.

### Personal questionnaire

Personal questionnaire comprised demographic data, length of service in LIC, operational stressors, domestic stressors, unit stressors, physical environment, living conditions, officer–man relationship and administrative aspects such as leave, welfare, posting/tenures, mail, rest and recreation, etc. It contained 46 structured questions and 8 open-ended questions.

### Carroll Rating Scale for Depression (CRSD)

The Carroll Rating Scale for Depression^20^ (CRSD) was developed as a self-rating instrument for depression, closely matching the information content and specific items of the Hamilton Depression Rating Scale (HDRS). The CRSD has acceptable face validity, reliability, internal consistency, as well as concurrent validity with HDRS and Beck Depression Inventory. It can be used to assess the severity of depression. It can also be used as a screening instrument with a score of 10 as a cut-off point.

### Michigan Alcoholism Screening Test (MAST)

The Michigan Alcoholism Screening Test^21^ (MAST) is a 24-item screening instrument designed to identify and assess alcohol abuse and dependence. The MAST has a high internal consistency with an alpha coefficient of 0.95. Some critics of self-report instruments suggest that their reliability is reduced by the reluctance of those with problem drinking to accurately report the extent and nature of their alcohol use. The relationship between social desirability, as measured by the Deny–Bad scale of the Crowne–Marlow Social Desirability Scale, and scores on the MAST indicated that although there was some relationship between the two measures, the correlations were small. Further analysis controlling for individual Deny-Bad scores found little change in MAST scores, indicating that any tendency to deny undesirable characteristics does not materially affect the validity of the MAST as a screening instrument.

### General Health Questionnaire (GHQ)

The General Health Questionnaire^22^ (GHQ) is a self-administered screening test, which is sensitive to the presence of psychiatric disorders in individuals presenting in primary care settings and non-psychiatric clinical settings. The GHQ is not designed to detect symptoms that occur with specific psychiatric diagnoses, rather, it provides a measure of overall psychological health or wellness. The GHQ has reasonable test–retest reliability. The GHQ has both content validity and construct validity. In the present study the shorter version containing 12 items—the GHQ-12—was used. The GHQ-12 has a sensitivity of 89% and specificity of 80%. The GHQ has been translated into 38 languages and used in diverse cultural groups. As it is primarily concerned with the detection of ‘psychological illness’, the items appear to have cross-cultural relevance despite cultural variations in the expression of mental illness.

### State-Trait Anxiety Inventory (STAI)

The State-Trait Anxiety Inventory^23^ (STAI) is a self-report assessment device which includes separate measures of state and trait anxiety. State anxiety reflects a ‘transitory emotional state or condition of the human organism that is characterized by subjective, consciously perceived feelings of tension and apprehension, and heightened autonomic nervous system activity’. State anxiety may fluctuate over time and can vary in intensity. In contrast, trait anxiety denotes ‘relatively stable individual differences in anxiety proneness…’ and refers to a general tendency to respond with anxiety to perceived threats in the environment. The scale has acceptable reliability and validity.

### Perceived Stress Questionnaire (PSQ)

The Perceived Stress Questionnaire^24^ (PSQ) is a 30-question self-rating scale for assessment of stress in general (past year or two) and recent (past month) forms. The recent form was utilized in the present study. The recent PSQ has acceptable validity and reliability. Recent PSQ scores are associated with trait anxiety, Cohen's perceived stress scale, depression, self-rated stress and stressful life events.^24^

### Impact of Events Scale (IES)

The Impact of Events Scale^25^ (IES) was developed to measure current subjective distress related to a specific event. The IES scale consists of 15 items, 7 of which measure intrusive symptoms, 8 tap avoidance symptoms, and combined, provide a total subjective stress score. Both the intrusion and avoid-ance scales have displayed acceptable reliability (alpha of 0.79 and 0.82, respectively), a split-half reliability for the whole scale of 0.86 and a test–retest reliability of 0.87 for the total stress scores, 0.89 for the intrusion subscale, and 0.79 for the avoidance subscale. The IES has also displayed the ability to discriminate a variety of traumatized groups from non-traumatized groups.

### Multidimensional Fatigue Inventory (MFI)

The Multidimensional Fatigue Inventory^26^ (MFI) is a self-report instrument containing 20 statements which cover different aspects of fatigue. These 20 items are organized in five scales. Each scale contains four items. The scales are balanced to reduce the influence of response tendencies as much as possible; each scale contains two items indicative of fatigue and two items contra-indicative of fatigue. The five scales measure general fatigue, physical fatigue, reduced activity, reduced motivation and mental fatigue.

### Locus of Control (LOC) scale

Locus of Control (LOC) scale^27^ is a construct embedded in social learning theory of Rotter. It stresses the role of expectancy and reinforcement value related with the outcome of behaviors or events. The scale has acceptable internal consistency, temporal stability and validity.

### Satisfaction with Life Scale (SWLS)

The Satisfaction with Life Scale^28^ (SWLS) consists of 5 items that are completed by the individual whose life satisfaction is being measured. Administration is brief—rarely more than a few minutes—and can be completed both by interview (including phone) and by paper-and-pencil response. The internal consistency of the SWLS is adequate with alpha coefficients repeatedly exceeding 0.80. Similarly, test–retest reliabilities have been generally acceptable. Factor analysis of the 5-item scale showed that it represented a single factor, which accounted for 66% of the variance in the instrument. Item to total score correlations have ranged from 0.57 to 0.66. The original validation studies correlated the SWLS with 10 other measures of subjective well-being. Most measures correlated at an r=0.50 or higher for each of the two samples from the original work. Subsequent studies have found comparable or higher correlations.

### The Hindi PEN inventory (PEN)

The Hindi PEN inventory^29^ (PEN) measures four dimensions of personality, viz. psychoticism (P) tendency or propensity to develop psychotic symptoms under stress; extraversion (E) (Eysenckenian model of introversion–extraversion dimension, defining extroversive as social, mixing, outgoing); neuroticism (N) or emotional instability defined as the propensity to develop and sustain neurotic symptoms under stress, and Lie scale (L) or tendency to give socially desirable responses in place of real responses. Higher the score greater the strength of that particular dimension of personality.^29^

The instruments were scored as per the test booklets. Data were tabulated and subjected to statistical analysis using SPSS 13.0 for Windows.

## RESULTS

The mean age of subjects in LIC and Other areas was 29.89 (SD=5.88; range 21–51) years and 30.37 (SD=6.31; range 20–50) years, respectively. All the subjects were male. The mean length of service of subjects in LIC and other area was 10.87 (SD=5.54) years and 11.12 (SD=6.03) years, respectively. There were no statistically significant differences between the two groups with regard to age, length of service, duration of stay in present location, rank, education and marital status. The mean duration of present deployment in LIC and Other areas was 19.46 (SD=12.81) months and 19.88 (SD=11.20) respectively. However, it was observed that a small number of men had served for a continuous period of five years or even more in various LIC/difficult areas. The vast majority of the subjects (82.9%) felt that 2 years should be the optimal duration of the tour of duty in LIC. Eleven per cent of the respondents felt that the tenure should be one year, while only 5.99% opted for 3-year tenure.

Table 1 depicts some of the major determinants of motivation and morale. A feeling of insecurity with regard to families back home, lack of societal support, adverse publicity in the media, hostile attitude of human rights groups, lack of cooperation/hostility on the part of the local population, dissatisfaction with regard to the financial compensation, difficulties encountered in rail travel, and a sense of disgust towards a corrupt polity were some of the factors affecting morale.

**Table 1:** Negative and positive determinants of morale

S. No. Negative determinants		Subjects in LIC (*n*=568)(%)	Subjects in Other areas (*n*=568) (%)	Chi-square value	P
*Operational Factors*					
1.	Anger at fighting with constraints	79.75	59.68	54.19	<0.01 S
2.	Anger at public admonishment	77.11	68.13	11.52	<0.01 S
3.	Bitterness at inability to deal with 'jamayatis'	59.33	36.62	15.73	<0.01 S
4.	Ambiguity regarding aim	26.94	27.11	0.00	>0.95 NS
5.	Feeling of uncertainty	24.82	17.43	9.32	<0.01 S
6.	Futility of counter-insurgency operations	23.77	15.67	11.70	<0.01 S
7.	Fear of ever present danger/unexpected attack	20.07	13.38	9.13	<0.01 S
*Social Factors*					
1.	Financial dissatisfaction	54.23	58.45	2.06	>0.10 NS
2.	Lack of cooperation/hostility from local population	53.17	38.56	24.42	<0.01 S
3.	Lack of societal support	50.35	46.66	1.55	>0.20 NS
4.	Disgust at a corrupt polity	47.71	46.12	0.28	>0.50 NS
5.	Adverse publicity in media	43.49	31.87	16.33	<0.01 S
6.	Difficulties in railtravel	36.09	45.95	11.41	<0.01 S
7.	Denial of leave even in emergency	35.39	36.80	0.24	>0.50 NS
8.	Hostile attitude of human rights groups	32.92	19.54	26.28	<0.01 S
9.	Feeling of insecurity regarding families	31.51	40.14	9.19	<0.01 S

	Positive determinants				

1.	Receiving mail regularly	81.16	80.63	0.05	>0.70 NS
2.	Regimental spirit	80.28	81.51	0.28	>0.50 NS
3.	Receiving all entitlements of pay and allowances	79.05	78.70	0.03	>0.70 NS
4.	State of living accommodations	76.76	82.21	5.19	<0.05 S
5.	Recreational facilities	70.25	80.28	15.36	<0.01 S
6.	Leave granted in emergency	63.03	61.44	0.30	>0.50 NS
7.	Fear of letting down family prestige	48.24	58.98	13.17	<0.01 S

S=Significant; NS=Not significant

In the operational context the factors exercising a negative impact included ambiguity with regard to aim, feelings of uncertainty, feeling of fighting a futile war with no benefits to the country, fear of ever-present danger/attack from unexpected quarters, feelings of anger/frustration at fighting with ‘one arm tied behind the back’ and anger/bitterness at not being able to deal with the unarmed but vicious ideologues/ motivators/ financiers of militants, the ‘jamayatis’ who were blatantly misusing religious institutions such as ‘madrasas’ in their antinational activities. These negative factors were counter-balanced by factors such as regimental spirit, group cohesiveness and the feeling of organizational support which contributed to high morale despite the dangers and hardships involved in LIC.

Results of the psychological tests indicated that compared to personnel from other areas, respondents from LIC area had significantly higher scores on CRSD, MAST, GHQ, IES, and general fatigue, physical fatigue, and mental fatigue subscale of the MFI. There are no significant differences in personality traits on PEN inventory. On the LOC scale there was no significant difference in the scores obtained by the two groups. It was seen that the majority of soldiers had external locus of control in both operational and peace locations indicating high adaptability. An extremely important finding was that there was no significant difference in the scores on the SWLS scale in personnel from LIC and Other areas, despite the former group obtaining significantly higher scores on CRSD, GHQ, MAST, IES and MFI (Table 2). Personnel in LIC had obtained significantly higher total scores as well as scores on intrusive and avoidance scale of IES (Table 2). Further analysis of the scores of the IES on dimensions revealed that as compared to individuals in Other areas, significantly less soldiers in LIC scored in the subclinical range; significantly more individuals in LIC scored in the mild and moderate range; while only very few personnel in LIC and none in Other areas had scores in the severe range (Table 3). Analysing the scores of the CRSD, MAST and GHQ as screening tests with cut-off points of >10, >5 and >2, respectively, it was found that individuals in LIC had significantly higher depression, alcohol abuse and psychiatric distress compared to those in other locations (Table 4). Further analysis of the psychological test results showed that adverse psychological effects were significantly related to the level of intensity of LIC (Table 5) and the length of service in LIC (Table 6).

**Table 2:** Results of psychological tests

S. No. Tests		Subjects in LIC		Subjects in other areas		Mann-Whitney	P
		Mean	(SD)	Mean	(SD)	U test z value	
1.	*Carroll Rating Scale for Depression*	8.68	(5.66)	6.54	(3.36)	−6.366	<0.000 S
2.	*Michigan Alcoholism Screening Test*	4.05	(4.57)	2.52	(3.06)	−5.177	<0.000 S
3.	*General Health Questionnaire*	2.13	(2.27)	1.41	(1.65)	−5.168	<0.000 S
4.	*State-Trait Anxiety Inventory*				
	State anxiety	35.69	(7.94)	35.36	(7.73)	−0.772	<0.440 NS
	Trait anxiety	33.99	(7.24)	33.77	(6.79)	−0.591	<0.555 NS
5.	*Perceived Stress Questionnaire*	73.44	(7.56)	73.27	(8.54)	−1.165	<0.244 NS
6.	*Impact of Events Scale*				
	Intrusive	3.67	(4.73)	2.72	(4.13)	−4.851	<0.000 S
	Avoidance	10.71	(6.73)	8.77	(7.33)	−4.940	<0.000 S
	Total	14.28	(9.12)	11.47	(9.00)	−4.788	<0.000 S
7.	*Multidimensional Fatigue Inventory*				
	General fatigue	8.44	(3.29)	7.51	(3.08)	−4.992	<0.000 S
	Physical fatigue	7.90	(4.04)	6.91	(3.34)	−3.501	<0.000 S
	Reduced Activity	9.16	(3.67)	8.79	(2.81)	−0.929	<0.353 NS
	Reduced Motivation	7.46	(2.88)	7.11	(2.42)	−1.303	<0.193 NS
	Mental fatigue	7.0	(2.90)	6.78	(3.13)	−2.418	<0.016 S
8.	*Locus of Con trol scale*	18.83	(9.69)	19.38	(8.07)	−1.598	<0.110 NS
9.	*Hindi PEN Inventory*				
	Psychoticism	2.98	(2.78)	3.13	(2.10)	−1.522	<0.128 NS
	Extra version	12.50	(2.69)	12.69	(2.55)	−0.783	<0.434 NS
	Neuroticism	4.48	(3.55)	4.84	(3.66)	−1.493	<0.135 NS
	Lie	11.00	(2.67)	11.26	(2.94)	−1.829	<0.067 NS
10.	Satisfaction With Life Scale	22.71	(7.93)	22.47	(7.56)	−0.928	<0.353 NS

S=Significant; NS=Not significant

**Table 3:** Comparison of Impact of Events Scale scores of subjects in LIC and Other areas

Impact of Events Scale scores	Subjects in LIC areas	Subjects in Other areas	Chi-square value	P
0–8 (subclinical)	24.65%	42.78%	41.78	<0.01 S
9–25 (mild)	66.37%	53.35%	20.06	<0.01 S
26–43 (moderate)	8.45%	3.87%	9.65	<0.01 S
44+ (severe)	0.53%	0.0%	-	-

S=Significant; NS=Not significant

**Table 4:** Comparison of screening psychological tests results of subjects in LIC and in other areas

Psychological tests	Subjects in LIC areas (*n*=568)	Subjects in Other areas (*n*=568)	Chi-square value	P
Carroll Rating Scale for Depression Score >10 Michigan Alcoholism	30.99%	9.16%	84.37	<0.01 S
Screening Test Score >5 General Health	29.93%	10.74%	64.56	<0.01 S
Questionnaire Score >2	33.28%	20.60%	23.19	<0.01 S

S=Significant; NS=Not significant

**Table 5:** Comparison of screening psychological tests results of subjects in very active LIC and less active LIC areas

Psychological tests	Subjects in very active LIC area	Subjects in less active LIC area	Chi-square value	P
Carroll Rating Scale for Depression Score >10 Michigan Alcoholism	39.48%	23.23%	17.50	<0.01 S
Screening Test Score >5 General Health	38.01%	24.35%	16.13	<0.01 S
Questionnaire Score >2	39.85%	27.27%	8.32	<0.01 S

S=Significant; NS=Not significant

**Table 6:** Relationship of screening psychological test results to length of service in LIC operations

Psychological tests	Subjects in with <1 year service in LIC	Subjects in with >1 year service in LIC	Chi-square value	P
Carroll Rating Scale for Depression Score >10 Michigan Alcoholism	16.77%	36.91%	22.36	<0.01S
Screening Test Score >5 General Health	16.17%	36.41%	22.81	<0.01S
Questionnaire Score >2	21.56%	38.16%	14.63	<0.01S

S= Significant; NS=Not significant

## DISCUSSION

Gabriel^30^ has noted that: ‘Nations customarily measure the “costs of war” in dollars, lost production, or the number of soldiers killed or wounded.’ But ‘rarely do military establish–ments attempt to measure the costs of war in terms of individual suffering. Psychiatric breakdowns remain one of the most costly items of war when expressed in human terms.’ Indeed, for the combatants in every major war fought in the twentieth century, there has been a greater possibility of becoming a psychiatric casualty than of being killed by enemy fire. It is essential to acknowledge that good ends have been and will continue to be accomplished through combat. Few individuals will deny the need for combat in World War II. Around the world the price of civilization is being paid every day by military units in LIC/peace-keeping operations and paramilitary and police forces that are forced to engage in close combat. There have been and will continue to be times and places where combat is unavoidable, but when a society requires its security forces to participate in combat, it is essential to fully comprehend the magnitude of the inevitable psychological toll.^31^ Denial of the psychological consequences of combat may be perilous.

The most strategic resource that India has in LIC is the young officer and soldier of the Indian Army. He is facing the brunt of the bullets and the wrath of the militants in an environment that is at odds with nature and politics. If casualties are an indicator of morale, guts and frontline leadership, then the soldiers pass the test with distinction.^2^ Surprisingly, very few studies have evaluated their psycho–logical state, which is addressed here. The present tour of duty of the soldier in LIC lasts 3 years. That this tenure is rather unpopular is evident from the fact that only 5.99% of the respondents preferred it. When compared to one-year service for soldiers in Vietnam, Iraq and Afghanistan, this prolonged tenure is unfair by all accounts. Compounding the problem is the fact that in some cases the long tenure in LIC is either preceded or followed by another ‘difficult’ tenure. It is well established that long tenures produce combat fatigue and an early ‘burn-out’. With about 265 days of hands-on service in a calendar year, troops are known to average about 16 hours a day in active operations with five hours of sleep and one off-day in eleven days. By any calculation this is mentally and physically stressful. When accompanied by an indifferent habitat (especially during winters), inadequate food and recreation facilities, under strength companies, uncertainty, tension, isolation, lack of work appreciation and public admonishments and common frustrations, the cumulative effects can be debilitating.^2^,^18^,^19^

The operational factors most often cited by the personnel as having a negative impact on morale was anger at fighting with constraints, which along with bitterness at inability to deal with ‘jamayatis’, ambiguity regarding aim, feeling of uncertainty and feeling of futility about LIC (Table 1), are inherent parts of such operations and are on expected lines. In fact the low figure for ambiguity regarding aim, feeling of uncertainty and feeling of futility is indicative of good morale. However, anger at public admonishment mentioned by 77.11% of respondents is definitely a matter of concern because in the surcharged atmosphere of LIC this can be the spark that starts a fire. Obviously preventive measures should be taken to address this problem. An important point was that fear of ever-present danger/unexpected attack was endorsed by only 20.07% respondents indicating that despite all the adversity the morale of the troops is high and that they are ready for every eventuality. The negative impact on morale of socio-political factors such as lack of societal support, adverse pro-paganda by the media/human rights activists, uncooperative/hostile attitude of the local population and a sense of disgust at the rampant corruption and moral degradation, which have been highlighted by earlier workers^1^,^19^,^32^,^33^ were confirmed in the present study, too (Table 1).

The aforesaid operational factors, which have a negative impact on the morale, may also affect the mental health of soldiers as indicated by our findings. While this may not immediately manifest as syndromal mental disorders, they do render the troops more vulnerable psychologically and may have long-term consequences. They are inherent in any LIC, as the Americans learnt the hard way in their defeat and humiliation in Vietnam.^1^ The experience gained by the Indian soldier over the years in LIC operations has lead to better weaponry and modification of tactics, with consequently growing successes in recent years. The importance of such time-tested concepts of soldiering such as regimental spirit, discipline, group cohesiveness, identification with the organization and loyalty to family/clan/tradition are even today potent motivators for men going into battle(1,2,19 has been confirmed once again by the findings of the present study (Table 1).

Though LIC are less stressful compared to combat, recent reports have shown links between exposure to peacekeeping-related events in Kosovo and an increase in physical symptoms, use of aggressive tactics, reduced sleep, increased number of days lost because of illness, and increased alcohol use, depression and PTSD symptoms.^11^ Soldiers deployed as peacekeepers can experience anxiety, frustration and helplessness from their peacekeeping role and can be exposed to events that are potentially traumatizing, e.g. mass killing, injured civilians, and landmines. Although the majority may cope well with the demands of a peacekeeping deployment, exposure to peacekeeping stressors is also associated with PTSD, depression and problems with aggression.^7^,^14^ An earlier Indian study^18^ also reported that use of alcohol was significantly higher in LIC (never users: LIC 3.5%, Others 34.5%; Occasional users: LIC 95.5%, Others 64%). Further, troops in LIC were significantly more frustrated (LIC 45.7%, Others 13.5%), easily upset (LIC 11.1%, Others 2%), felt isolated (LIC 84.9%, Others 72.5%), tense (LIC 31.1%, Others 11.5%), depressed (LIC 22.1%, Others 0%) and had decreased work efficiency (LIC 43.2%, Others 5.5%) as compared to troops deployed in similar terrain and climate but not in LIC operations. The significantly higher scores on CRSD and MAST in soldiers in LIC are thus in agreement with the literature. That some soldiers do have problems with aggression is proved by occasional media reports of soldiers running berserk and shooting their colleagues or superiors on trivial issues.

The finding of significantly high GHQ scores in personnel after deployment in LIC operations is in agreement with earlier studies.^5^,^10^,^18^ An earlier Indian study reported that psychiatric morbidity, both outdoor and indoor, was almost three times higher for troops deployed in LIC operations (rate of OPD cases in LIC 5.33, Others 1.66, p=0.009; rate of indoor cases in LIC 4.13, Others 1.33; p=039).^18^ Hoge *et al.*^5^ studied US infantry soldiers using screening instruments in an anonymous survey either before (*n*=2530) or 3–4 months after their deployment (*n*=3671) in Iraq or Afghanistan. The percentage of study subjects whose responses met the screening criteria was significantly more after deployment for depression, anxiety, PTSD and alcohol problems. This is broadly in agreement with our findings except that anxiety was not increased in our sample. Evaluation of 200 UK servicemen with the GHQ-28 before and after a 6-month tour of duty in Northern Ireland^10^ revealed high baseline levels of psychological morbidity, and three times increase in caseness after the operational tour which is similar to our findings.

PTSD is the most widely reported disorder related to combat as well as peacekeeping duties in both lay accounts and published research.^5^ Surprisingly, no Indian study has addressed this aspect of LIC, though clinical experience suggests that PTSD is uncommon in Indian soldiers exposed to LIC or combat. A survey conducted by Litz *et al.*^9^ of 3461 active duty personnel deployed in Somalia 5 months after their return to the US revealed that 18% had PTSD. PTSD symptom severity was best predicted by the war zone stress and frustrations with peacekeeping. The best negative predictors of PTSD were the generic rewards of soldiering, i.e. pride in serving the country, group cohesion and confidence in the order and structure of the military. The finding of 8.98% of our soldiers scoring in the moderate to severe range of the IES (Table 4) is in agreement with the above; although it must be emphasized that as opposed to the 6–12 months tour of the US soldiers our sample had an average tenure of 19.5 months in LIC area at the time of testing. This finding underlines the inherent resilience of the Indian soldier. The better resilience^1^,^2^,^34^ could be due to group cohesiveness, good leadership and high morale indicated from the results of the questionnaire. Moreover, unlike western troops our soldiers are operating in their own land and to protect the integrity of the country and so the mission is clearly defined. Further there are firm instructions to minimize collateral damage. As a result there is lack of participation in excessive violence and consequently an absence of guilt which may explain why Indian soldiers are able to adapt favorably to their war zone experiences.

Fatigue is a normal, everyday experience that most individuals report after inadequate sleep or rest, after physical exertion and mental effort. The finding of significantly higher scores on the general fatigue, physical fatigue, reduced motivation and mental fatigue subscale of the MFI by personnel in LIC is of course on the expected lines. However, it is interesting to note that our sample had much lower scores than military recruits in training and in barracks.^25^ On LOC scale majority of soldiers had external locus of control in both LIC and Other locations indicating high adaptability which is a useful characteristic in soldiers. On the PEN also there were no significant differences in the scores in LIC and peace areas. The low scores on neuroticism and high scores on lie scales is in agreement with an earlier study.^35^

The finding that soldiers in LIC had significantly higher depression, alcohol abuse and psychiatric distress compared to those in Other locations (Table 4) and that these adverse psychological effects were significantly related to the level of intensity of LIC (Table 5) and the length of service in LIC (Table 6) is in agreement with earlier studies.^8^,^9^,^14^,^18^,^19^ This indicates the need to sensitize medical and administrative authorities about these problems so that preventive measures can be instituted. An extremely important finding was that there was no significant difference in the scores on the SWLS scale in personnel from LIC and other area, despite the former group obtaining significantly higher scores on CRSD, GHQ, MAST, IES and MFI (Table 2). This finding again confirms the belief that despite the adversities and hardships of service in LIC areas the morale of the troops is high and that the general attitude towards life was optimistic.

Thus, it must be said the results of the psychological tests in our study were on the whole very satisfying. There was no increase in anxiety, stress, psychoticism, and neuroticism, and most importantly both groups of personnel had similar SWLS scores. It undoubtedly speaks volumes about the high morale, astute leadership and most importantly the resilience of the Indian soldier. This should not, however, lull the nation into a state of self-congratulatory complacency. The redoubtable Indian soldier cannot be expected to withstand the stresses of LIC indefinitely unscathed. The results of the psychological tests indicate that some of the soldiers are depressed and psychologically unwell, and this is related not only to the intensity of LIC but also to the length of time spent in these areas. Further, on the IES 8.45% were placed in the moderate range and 0.53% were placed in the severe range (Table 3). This may well be a portent of things to come and indicate the need for instituting immediate remedial and preventive measures.

LIC are primarily junior officers'/NCOs' wars. There is a need for a ‘thinking soldier’. The top-down military approach of a conventional war may not work in such operations. The soldiers' self-image and professionalism are important factors in preventing emotions ruling the mind and determining behaviour. The importance of adequate mental conditioning through indoctrination, training in public relations, and strengthening junior leadership at NCO level cannot be overemphasized.^2^ Finally, it needs to be realized that in the light of recent politico-military developments at the inter-national level and particularly in our neighborhood, the counter-insurgency duties for the armed forces are here to stay. Though welfare measures like liberal leave, improved leave travel facilities and enhanced monetary benefits for difficult tenures have helped to boost the morale of troops in recent years, more needs to be done. It may be mentioned here that simple measures such as curtailment of tenures/regular rotation have been found to be extremely effective in other countries facing similar situations.

Lastly, the findings of the study emphasize the need to further increase the availability of mental health services in the security forces. In the military, there are unique factors that contribute to resistance from seeking such help, particularly concern about how a soldier will be perceived by peers and by the leadership. A recent US study^5^ of soldiers deployed in Iraq and Afghanistan reported that concern about stigma was disproportionately greatest among those most in need of psychiatric help. Soldiers whose responses were scored as positive for a mental disorder were twice as likely as those whose responses were scored as negative to show concern about being stigmatized and about other barriers to mental health care. Somehow psychiatric treatment in the security forces has acquired a dirty label and stigmatizes the individual seeking help from a psychiatrist. There is an urgent need for all, most importantly the commanders, to move away from this thinking. Innumerable studies attest the fact that troops who operate for protracted periods under stressful conditions are bound to suffer from psychological distress as well as psychiatric disorders. This is very natural and there is no need to look upon psychiatric problems with alarm and hide them in the closet.^2^

### Limitations

1. As the present study was carried out in operational areas, only self-rated rating scales were used though observer rated instruments are more reliable.
2. In the present study for sake of confidentiality the unit and trade of the subject was not recorded. In subsequent studies it would be informative to see if the stress on fighting soldiers and supporting troops is different.
3. Only male subjects were included in the present study. In subsequent studies females should also be included.

## CONFLICT OF INTEREST

None
